# Diabetic Polyneuropathy and Physical Activity in Type 1 Diabetes Mellitus: A Cross-Sectional Study

**DOI:** 10.3390/jcm12206597

**Published:** 2023-10-18

**Authors:** Simona Zaccaria, Pasquale Di Perna, Laura Giurato, Chiara Pecchioli, Patrizia Sperti, Flavio Arciprete, Alessandra Del Grande, Isabella Nardone, Sium Wolde Sellasie, Cesare Iani, Luigi Uccioli

**Affiliations:** 1 Division of Endocrinology and Diabetes, CTO Andrea Alesini Hospital, Department of Biomedicine and Prevention, University of Rome Tor Vergata, 00133 Rome, Italy; simona.zaccaria@students.uniroma2.eu (S.Z.); pasquale.diperna@aslroma2.it (P.D.P.); laura.giurato@aslroma2.it (L.G.); chiara.pecchioli@aslroma2.it (C.P.); patrizia.sperti@aslroma2.it (P.S.); isabella.nardone@students.uniroma2.eu (I.N.); sium.woldesellasie@students.uniroma2.eu (S.W.S.); 2 Division of Neurology, Sant’Eugenio Hospital, 00144 Rome, Italy; flavio.arciprete@aslroma2.it (F.A.); alessandra.delgrande@aslroma2.it (A.D.G.); cesare.iani@aslroma2.it (C.I.)

**Keywords:** diabetic polyneuropathy, type 1 diabetes, physical activity, IPAQ

## Abstract

Background: The purpose of this study is to access whether a personal attitude to physical activity (PA) may influence the appearance of diabetic polyneuropathy (DPN) patients with well-controlled type 1 diabetes mellitus. Methods: Ninety patients attending the diabetes technology outpatient clinic were enrolled. DPN was investigated according to the Toronto consensus diagnostic criteria. PA was assessed using the International Physical Activity Questionnaire. Results: PA was low in 21.1%, moderate in 42.2% and high in 36.7% of patients. According to Toronto criteria, we defined two categories: the first one with DPN absent or possible (57 (63.3%)) and a second one with DPN certain or probable (33 (36.7%)). The χ^2^-test of the PA groups and the DPN categories showed a statistically significant difference (*p* < 0.001), with less neuropathy in patients belonging to the group of moderate/high PA. Exposure to a minimum of 600 MET minutes/week was protective factor against the onset of DPN (odd ratio 0.221, c.i. 0.068–0.720, *p* = 0.012). Conclusions: This study suggests that DPN is less present in type 1 diabetic patients with good metabolic control and a good personal habit of PA. Moderate-to-vigorous PA of at least 600 MET minutes/week might be a protective factor against DPN.

## 1. Introduction

The increasing average age of the population is accompanied by an increasing number of years spent living with diabetes mellitus and its complications. To organise prevention and intervention programmes, it is necessary to know the burden of diabetic complications, and diabetic neuropathy is one of the most complex long-term complications, which can be evaluated in terms of prevalence, incidence and severity.

Epidemiological data for diabetic neuropathy available in the literature are influenced by the characteristics of the studies (sampling methods), the population studied and the diagnostic approach. The incidence of neuropathy is 6100 per 100,000 person-years in individuals with type 2 diabetes mellitus (T2DM), and 2800 per 100,000 person-years in those with type 1 diabetes mellitus (T1DM). [1] By contrast, the prevalence of neuropathy is similar in T2DM (8–51%) [2,3,4,5] and in T1DM (11–50%) [1,4,5,6].

The most typical and common form of diabetic neuropathy (more than 80% of cases) is sensorimotor polyneuropathy (DPN), of which painful diabetic neuropathy (PDN) is a variant. DPN has recently been redefined as a length-dependent distal symmetric sensorimotor polyneuropathy, characterised by the loss of sensation and distal motor function resulting from exposure to chronic hyperglycaemia and various cardiovascular risk factors that cause metabolic and microvascular damage.

There is no specific treatment for this complication; however, management of clinical conditions such as hyperglycaemia, hypertension, dyslipidaemia, body weight and more (in general, the acquisition of a healthy lifestyle including healthy eating and regular physical activity) may influence its severity and progression [7,8].

One of the mechanisms by which physical activity (PA) helps to slow the progression of the disease is related to its capability to modulate inflammation [9,10] and to reduce mean glucose levels [11,12]. Studies have shown that long-term physical activity is associated with less inflammation, with higher levels of exercise correlating to lower counts of white blood cells, neutrophils and C-reactive protein levels [10].

A structured exercise programme is also inversely associated with levels of glycated haemoglobin that are independent of body weight loss; an average reduction in glycated haemoglobin also depends on the intensity of PA [11,12].

The reason why exercise has historically been discouraged in people with DPN is that, in the presence of sensory loss associated with an impaired balance and an unstable gait, it may contribute to the development of foot ulcers, infections and lower limb amputations [13].

These generic indications were superseded only after the observation that diabetic patients with DPN and sensory loss who exercised through weight-bearing activities did not have an increased incidence of foot ulcers compared with a control group. In some cases, the incidence was even lower [14,15].

Indeed, evidence has not only indicated the safety of exercise in DPN [15] but, in addition, the role of exercise can be considered to be a therapeutic approach for its beneficial effects on neuropathy symptoms [16], gait function [17,18] and epidermal nerve fibre branching [19].

L Allet et al. [17] investigated the effect of exercise by studying two groups of type 2 diabetic patients with clinically diagnosed neuropathy; patients in the intervention group were subjected to a 12-week training programme consisting of two 60 min sessions per week based on exercises to improve gait and balance. The increase in gait, balance and muscle strength was statistically significant in the physical activity group.

Furthermore, Kluding et al. [19] observed that in patients with diabetic neuropathy, a 10-week exercise programme was able to improve pain levels and other symptoms assessed by MNSI score, such as glycaemic control. In addition, they also found an increased number of branches per fibre.

While the beneficial effects of PA on clinical diabetic neuropathy in type 2 diabetes mellitus are well known, the benefits of PA in type 1 diabetes mellitus are less defined. Therefore, we have tested a group of type 1 diabetic patients, who displayed relatively good metabolic control, with the aim to evaluate whether a personal attitude to regular physical activity, even performed independently of medical advice, is able to influence the appearance and severity of DPN.

## 2. Materials and Methods

### 2.1. Case Selection

This cross-sectional study was conducted from September 2022 to June 2023 at the diabetes technology outpatient clinic of the Diabetes and Endocrinology Department of the CTO Hospital (Rome, Italy). Ninety patients aged 18 to 65 years with a diagnosis of type 1 diabetes mellitus using either an insulin pump (64.4%) or insulin with a basal bolus schedule and continuous glucose monitoring (35.6%) were included.

Exclusion criteria were (a) a past or current history of severe or unstable psychiatric or physical illness (acute or progressive) that would prevent the patient from fully understanding the study or any condition that would pose an undue risk to the study procedures; (b) the presence of hemiparesis, myelopathy, cerebellar ataxia or other central nervous system diseases; (c) lower extremity arthritis or pain limiting exercise; (d) the presence of severe or acute cardiovascular disease that limits or contraindicates physical activity; (e) history or clinical signs of vestibular dysfunction; (f) history of angina or angina-like symptoms; (g) symptomatic postural hypotension; (h) previous or current presence of a plantar pressure ulcer on physical examination; (i) the presence of a high number of hypoglycaemic episodes in the holter glycaemic data, which contraindicate structured physical activity; (l) lack of consent for the processing of personal data. Informed oral and written consent were obtained from each patient.

### 2.2. Protocol

In accordance with usual clinical practice, the following data were collected from all enrolled patients:

- Biometric parameters (weight, height, BMI);
- Anthropometric parameters (age, gender);
- Physiological anamnesis (smoking, alcohol);
- Pathological history (duration of DM, presence of DM complications, hypertension);
- Clinical data (glycated haemoglobin (HbA1c), fasting glucose, total cholesterol, HDL, triglycerides, albumin-to-creatinine ratio (ACR), creatinine).

The presence of diabetic polyneuropathy was investigated by a careful assessment of symptoms (Michigan Neuropathy Screening Instrument questionnaire) [20], a neurological examination focused on the lower extremities, with particular emphasis on sensory function in the various modalities, including an inspection of the foot (DNI neurological examination scoring system) [21] and a nerve conduction study of the sural, motor peroneal and tibial nerves of the lower extremities.

Symptoms include decreased sensibility, positive neuropathic sensory symptoms (e.g., numbness, tingling, stinging, burning or constant pain) predominantly in the toes, feet or legs; symptoms were objectified using the Michigan Neuropathy Screening Instrument questionnaire (range score 0–13, cut off > 1).

In addition, signs may include the presence of foot deformities such as claw or hammer toes, dry skin, calluses, infections and ulcers, a symmetric decrease or reduction in the distal sensation and absent Achilles reflexes; these were assessed using the DNI (diabetic neuropathy index) with a range score of 0–8 and cut off > 2.

A nerve conduction study of the sural, motor peroneal and tibial nerves of the lower extremities was then performed, including nerve conduction velocity, motor action potential amplitude and latency.

Individuals were assigned to one of the four DPN groups according to the Toronto criteria:

- Possible sensorimotor diabetic polyneuropathy based on the presence of DPN symptoms or signs;
- Probable sensorimotor diabetic polyneuropathy based on the co-presence of symptoms and signs of DPN;
- Confirmed sensorimotor diabetic polyneuropathy because the patient has had abnormal findings in an abnormal nerve conduction study (defined as abnormal findings in at least two separate nerves, one of which should be the sural nerve) and a symptom or symptoms or sign or signs of DPN;
- Without sensorimotor diabetic polyneuropathy due to the absence of signs, symptoms and a negative nerve conduction study.

Physical activity has been assessed by completing the International Physical Activity Questionnaire (IPAQ). It was validated in 1997 as an instrument for monitoring different physical activity domains in adults aged 18 to 65 years, and it is also useful for comparing PA between countries and is currently being developed in 12 translations. There are two versions of the questionnaire: a short version and a long version. For this study, the long version was used and contains four questions on the patient’s demographic features (age, sex, education level, employment), six questions on the patient’s understanding of the questionnaire and twenty-seven questions on the PA performed. Concerning PA, it analyses (1) physical activity performed at work, (2) physical activity for active daily transportation, (3) physical activity performed for housework and family care, (4) physical activity during leisure time or sport, while a last domain investigates the time spends sitting.

The information about PA in the questionnaire was expressed in minutes per day and/or days per week [22].

All patients were classified into the following:

(1) Diabetics with high PA if they achieved a minimum total physical activity of 1500 MET minutes/week of vigorous-intensity activity on at least 3 days or who achieved a minimum total physical activity of 3000 MET minutes/week on 7 or more days of any combination of walking, moderate-intensity or vigorous-intensity activities;
(2) Diabetics with moderate PA if they are engaged in 20 min per day of vigorous activity for 3 or more days; if they engaged in 30 min per day of moderate activity or walking for 5 days or more; or, finally, if they achieved a minimum total physical activity of at least 600 MET minutes/week of walking, moderate activities or vigorous activities for 5 or more days;
(3) Inactive if the patient did not meet the criteria for the previous two categories.

In addition, in patients without known cardiovascular complications, we calculated the 10-year cardiovascular risk using a validated calculator for type 1 diabetics called the Steno type 1 risk engine. It combines 10 variables: age, sex, duration of diabetes, systolic blood pressure, low-density lipoprotein cholesterol, haemoglobin A1c, albuminuria, glomerular filtration rate, smoking and exercise, and it can predict cardiovascular disease, defined as a composite of fatal and non-fatal events of ischaemic heart disease, ischaemic stroke, heart failure and peripheral artery disease [23].

### 2.3. Statistical Analyses

Statistical analyses were performed with Jamovi version 2.3.28. Quantitative data were presented as mean (SD) and as median (range) when appropriate. Qualitative data were expressed as frequencies (number and percentage). Values of *p* < 0.05 were considered statistically significant. The χ^2^-test was used to analyse the association between frequencies of the variables. Analysis of variance (ANOVA) was used to explore the relationship between a continuous dependent variable and one categorical explanatory variable.

## 3. Results

During the period from September 2022 to June 2023, 90 patients belonging to the diabetes technology outpatient clinic were enrolled. Most of them had an insulin pump (64.4%), while the remaining had a basal bolus regimen and continuous glucose monitoring (35.6%).

The median age of the study participant was 49.0 years (41.0–55.8), and 47.8% of them were female. The mean duration of DM was 21.0 years (14.3–34.0), while the mean BMI was 23.6 kg/m^2^ (21.8–25.9).

Metabolic control at the time of the visit was good (HbA1c < 7.5% (58 mmol/mol)) in 51 (56.7%) participants, while 39 (43.3%) had glycated haemoglobin greater than 7.6% (60 mmol/mol).

Table 1 shows the demographic and clinical features of the study participants.

Of all the patients evaluated, 45.6% had diabetic complications such as retinopathy (28.9%), nephropathy (11.1%) and neuropathy (36.7%), while 26.7% of the sample had hypertension.

In 87 patients without known cardiovascular complications, the Steno type 1 risk engine was calculated; 3 patients from the sample were excluded because they were in secondary cardiovascular prevention. The 10-year CVD risk was low in 37 (42.5%), medium in 28 (32.2%) and high in 22 cases (25.3%).

Physical activity assessed by the International Physical Activity Questionnaire (IPAQ) was low in 19 (21.1%), moderate in 37 (41.1%) and high in 34 patients (37.8%).

According to physical activity (PA) (Table 1), no significant difference in age was observed between patients with low (51.0 years; 47.0–60.5), moderate (51.0 years; 39.0–56.0) and high (46.0 years, 38.0–50.0) PA (*p* = 0.125).

No differences in gender (*p* = 0.122), BMI (*p* = 0.053), smoking (*p* = 0.475), alcohol consumption (*p* = 0.798), educational level (*p* = 0.549), DM duration (*p* = 0.250), therapy (*p* = 0.404), DM complications such as retinopathy (*p* = 0.251), nephropathy (*p* = 0.382), carotid atherosclerosis (*p* = 0.508) and hypertension (*p* = 0.125) were found between the three groups of PA level.

**Figure 1:**
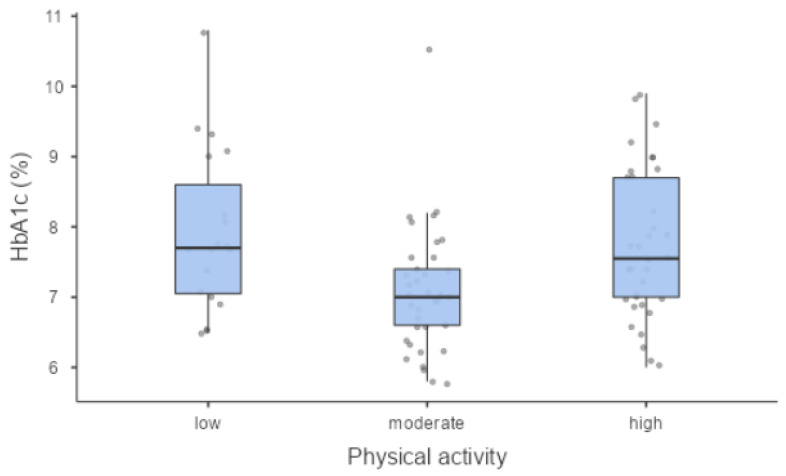
Metabolic control (HbA1c) was significantly different in PA groups.

**Figure 2:**
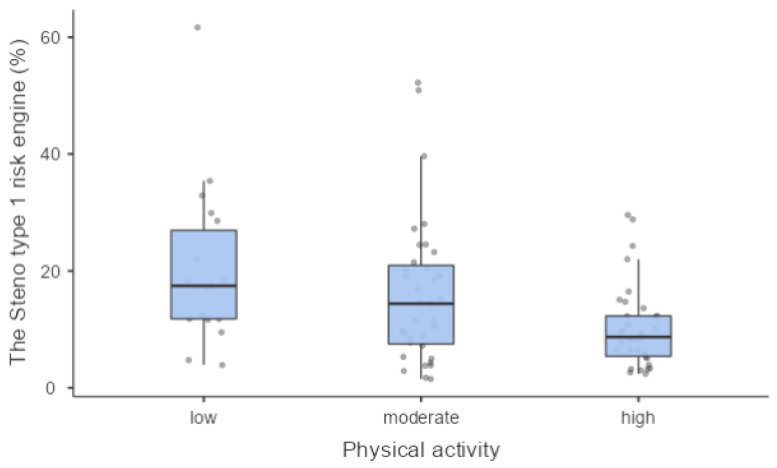
The Steno type 1 risk engine was significantly different in PA groups.

We found a statistically significant association between PA level and metabolic control expressed by glycated haemoglobin (7.70% (61 mmol/mol) (7.05–8.60 (54–70)); 7.00% (53 mmol/mol) (6.60–7.40 (49–57)); 7.55% (59 mmol/mol) (7.00–8.70 (53–72)) in low, moderate and high PA levels, respectively) (*p* = 0.004) (Figure 1) and between PA level and the 10-year CV risk (*p* = 0.045) (Figure 2).

**Figure 3:**
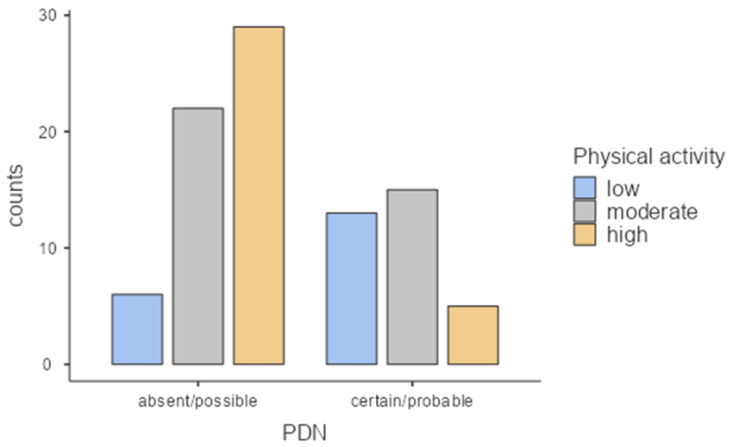
Relationship between diabetic polyneuropathy prevalence and the level of physical activity.

According to the Toronto Neuropathy Classification, patients were grouped in two groups: the first one with DPN absent or possible (n = 57 (63.3%)) and a second one with DPN certain or probable (n = 33 (36.7%)) (Figure 3).

When we analysed the association between neuropathy and the level of physical activity, we found 32.2% of diabetic patients without DPN in the high PA group and 14.4% of those with DPN in the low PA group. The χ^2^-test between PA groups and the diabetic DPN categories showed a statistically significant difference (*p* < 0.001). The percentage of each subgroup of DPN is detailed in Table 2.

A statistically significant relationship between PA and DPN was maintained in all groups when stratified by age, duration of disease, glycated haemoglobin, gender and hypertension.

Finally, binomial logistic regression was used to calculate the effect of physical activity on the prevalence of DPN, and the odds ratio for exposure of at least 600 MET minutes/week showed that this amount of PA was protective against the development of DPN (odds ratio = 0.221, confidence interval = 0.068–0.720, *p* = 0.012). This is in line with the main recommendation, which suggests a minimum of 150 min per week of moderate physical activity for type 1 diabetic patients [24].

**Figure 4:**
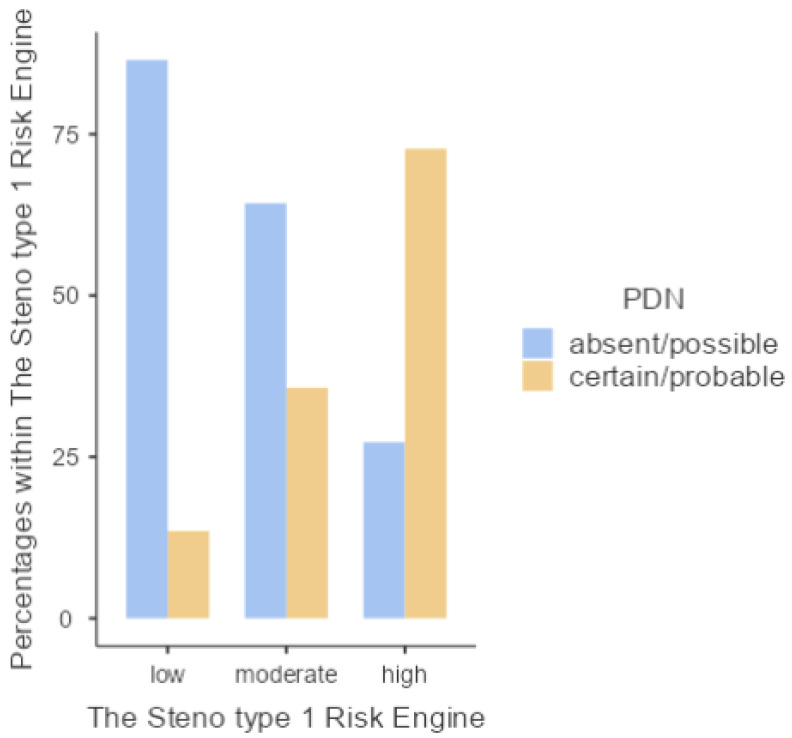
Levels of the Steno type 1 risk engine in relation to the presence of DPN.

When the association between a validated cardiovascular risk score for type 1 diabetic patients and the prevalence of diabetic polyneuropathy was analysed, 57.1% of the total sample with absent/possible DPN showed a low Steno type 1 risk engine, whereas 51.6% of the patients with certain/probable DPN showed a high CV risk (*p* < 0.001) (Figure 4).

## 4. Discussion

There’s evidence that low levels of PA are associated with the onset of diabetic neuropathy, but to date it has been shown that frequent structured physical activity does not correlate with a reduction in the prevalence of the disease, although it does help to improve neuropathic symptoms, balance and both motor and sensory neuromuscular parameters [1,19,25,26,27].

Balducci et al. demonstrated with their intervention study that long-term aerobic exercise training can modify the natural history of peripheral diabetic neuropathy or even prevent its onset in a period of 4 years [16].

Our study shows that a personal attitude to physical activity influences the appearance of DPN in a group of type 1 diabetic patients.

We used the International Physical Activity Questionnaire to assess the exercise of all patients at the time of visit. This questionnaire investigates the type of exercise routinely completed in a typical week.

There was no statistically significant difference in the three groups (low, moderate, and high PA) on age, sex, BMI, smoking, alcohol intake, educational level, duration of diabetes, therapy, retinopathy, nephropathy, carotid atherosclerosis and hypertension. This show us that the effect of exercise on DPN is not influenced by the most important confounding risk factors.

The groups were significantly different in terms of glycaemic haemoglobin and 10-year CVD risk; there was better glycaemic control in those with moderate PA compared to those with low and high PA.

Our study showed that moderate physical activity, reaching at least 600 MET minutes per week, seems to be a protective factor of diabetic neuropathy. This is underlined by the fact that according to several guidelines [24,28], most adults with diabetes should engage in 150 min or more of moderate intensity activity weekly spread over at least 3 days/week.

The 10-year CVD risk was significant lower in patients with high PA compared to patients with low PA, confirming the role of exercise in the prevention of cardiovascular complications [29].

The cardiovascular risk score that we calculated was statistically lower in patients who were physically active and higher in those who were inactive. This is in line with the study by Tesfaye S. and colleagues in which they observed that modifiable and non-modifiable risk factors, such as duration of diabetes, current glycated haemoglobin and smoking, all included in the Steno type 1 risk engine, were associated with the incidence of neuropathy [6].

The strength of our study is the homogeneity of the sample and having tests administered by the same operator.

On the other hand, our study has one important limitation, which is related to the sample size. Although well selected and homogeneous, the group of patients was relatively small. It obliged us to combine absent DPN with possible DPN and also combine certain DPN with probable DPN when classifying diabetic neuropathy according to the Toronto criteria.

## 5. Conclusions

In conclusion, this study suggests that a personal attitude towards physical activity may prevent or delay the onset of DPN, that moderate exercise of at least 600 MET minutes/week is a protective factor against the onset of DPN and that the main risk factors for CV diseases are the same for the development of polyneuropathy. Further studies will be necessary to confirm these data with a longitudinal aspect and a larger sample. To date, patients with type 1 diabetes mellitus should be educated to begin physical activity early, as well as insulin therapy, as part of their treatment plan and to prevent complications that may be associated with a long duration of the disease.

## Figures and Tables

**Table 1:** Demographic and clinical features of the study participants according to age, sex, BMI, smoking, alcohol, educational level, DM duration, DM therapy, HbA1c, DM complications, physical activity and the Steno type 1 risk engine.

Variables		Physical Activity			*p* Value
		Low 19 (21.1%)	Moderate 37 (41.1%)	High 34 (37.8%)	
Age (years)	49.0 (41.0–55.8)	51.0 (47.0–60.5)	51.0 (39.0–56.0)	46.0 (38.0–50.0)	0.125
Sex n (%)	male 47 (52.2)	9 (10.0)	24 (26.7)	14 (15.6)	0.122
	female 43 (47.8)	10 (11.1)	13 (14.4)	20 (22.2)	
BMI (kg/m^2^)	23.6 (21.8–25.9)	23.4 (20.9–24.9)	25.0 (22.9–28.5)	22.6 (21.3–23.9)	0.053
Smoking n (%)	24 (26.7)	7 (7.8)	8 (8.9)	9 (10)	0.475
Alcohol n (%)	70 (77.8)	14 (15.6)	30 (33.3)	26 (28.9)	0.798
Educational level n (%)	lower middle school 7 (7.8)	1 (1.1)	2 (2.2)	4 (4.4)	
	high school 50 (55.6)	10 (11.1)	19 (21.1)	21 (23.3)	0.549
	university 33 (36.7)	8 (8.9)	16 (17.8)	9 (10.0)	
DM duration (years)	21.0 (14.3–34.0)	23.0 (13.0–34.5)	24.0 (15.0–40.0)	19.5 (15.0–26.0)	0.250
DM therapy n (%)	CSII 58 (64.4)	14 (15.6)	21 (23.3)	23 (25.6)	
	Insulin basal-bolus 32 (35.6)	5 (5.6)	16 (17.8)	11 (12.2)	0.404
HbA1c % (mmol/mol)	7.35 (57) (6.82–8.07 (51–65))	7.70 (61) (7.05–8.60 (54–70))	7.00 (53) (6.60–7.40 (49–57))	7.55 (59) (7.00–8.70 (53–72))	0.004
DM complications n (%)	PDN absent/possible 57 (63.3)	6 (6.7)	22 (24.4)	29 (32.2)	<0.001
	certain/probable 33 (36.7)	13 (14.4)	15 (16.7)	5 (5.6)	
	Retinopathy 26 (28.9)	8 (8.9)	11 (12.2)	7 (7.8)	0.251
	Nephropathy 10 (11.1)	2 (2.2)	6 (6.7)	2 (2.2)	0.382
	Myocardial Infarction 3 (3.3)				
	Carotid artery atheromasia 16 (17.8)	4 (4.4)	8 (8.9)	4 (4.4)	0.508
Hypertension n (%)	24 (26.7)	7 (7.8)	12 (13.3)	5 (5.6)	0.127
Steno type 1 risk engine n (%)	low 37 (42.5)	3 (3.4)	14 (16.1)	20 (23)	
	moderate 28 (32.2)	9 (10.3)	10 (11.5)	9 (10.3)	0.045
	high 22 (25.3)	6 (6.9)	11 (12.6)	5 (5.7)	

**Table 2:** Prevalence of DPN in physical activity groups.

	Physical Activity
DPN absent/possible n. 57 (63.3%)	low n. 6 (6.7%)
	moderate n. 22 (24.4%)
	high n. 29 (32.2%)
DPN certain/probable n. 33 (36.7%)	low n. 13 (14.4%)
	moderate n. 15 (16.7%)
	high n. 5 (5.6%)

## Data Availability

Not applicable.

## References

[1] Feldman E.L., Callaghan B.C., Pop-Busui R., Zochodne D.W., Wright D.E., Bennett D.L., Bril V., Russell J.W., Viswanathan V. (2019). Diabetic Neuropathy. Nat. Rev. Dis. Primers.

[2] Franklin G.M., Kahn L.B., Baxter J., Marshall J.A., Hamman R.F. (1990). Sensory Neuropathy In Non-Insulin-Dependent Diabetes Mellitus. Am. J. Epidemiol..

[3] Partanen J., Niskanen L., Lehtinen J., Mervaala E., Siitonen O., Uusitupa M. (1995). Natural History of Peripheral Neuropathy in Patients with Non-Insulin-Dependent Diabetes Mellitus. N. Engl. J. Med..

[4] Dyck P.J., Kratz K.M., Karnes J.L., Litchy W.J., Klein R., Pach J.M., Wilson D.M., O’Brien P.C., Melton L.J. (1993). The Prevalence by Staged Severity of Various Types of Diabetic Neuropathy, Retinopathy, and Nephropathy in a Population-Based Cohort: The Rochester Diabetic Neuropathy Study. Neurology.

[5] Boulton A.J.M., Knight G., Drury J., Ward J.D. (1985). The Prevalence of Symptomatic, Diabetic Neuropathy in an Insulin-Treated Population. Diabetes Care.

[6] Tesfaye S., Chaturvedi N., Eaton S.E.M., Ward J.D., Manes C., Ionescu-Tirgoviste C., Witte D.R., Fuller J.H. (2005). Vascular Risk Factors and Diabetic Neuropathy. N. Engl. J. Med..

[7] Tesfaye S., Boulton A.J.M., Dyck P.J., Freeman R., Horowitz M., Kempler P., Lauria G., Malik R.A., Spallone V., Vinik A. (2010). Diabetic Neuropathies: Update on Definitions, Diagnostic Criteria, Estimation of Severity, and Treatments. Diabetes Care.

[8] Dyck P.J., Albers J.W., Andersen H., Arezzo J.C., Biessels G.-J., Bril V., Feldman E.L., Litchy W.J., O’Brien P.C., Russell J.W. (2011). Diabetic Polyneuropathies: Update on Research Definition, Diagnostic Criteria and Estimation of Severity. Diabetes Metab. Res. Rev..

[9] Loprinzi P.D., Hager K.K., Ramulu P.Y. (2014). Physical Activity, Glycemic Control, and Diabetic Peripheral Neuropathy: A National Sample. J. Diabetes Complicat..

[10] Loprinzi P.D., Ramulu P.Y. (2013). Objectively Measured Physical Activity and Inflammatory Markers Among US Adults with Diabetes: Implications for Attenuating Disease Progression. Mayo Clin. Proc..

[11] Boulé N.G., Haddad E., Kenny G.P., Wells G.A., Sigal R.J. (2001). Effects of Exercise on Glycemic Control and Body Mass in Type 2 Diabetes Mellitus. JAMA.

[12] Sigal R.J., Kenny G.P., Wasserman D.H., Castaneda-Sceppa C., White R.D. (2006). Physical Activity/Exercise and Type 2 Diabetes. Diabetes Care.

[13] (2006). American Diabetes Association; Standards of Medical Care in Diabetes–2006. Diabetes Care.

[14] Armstrong D.G., Lavery L.A., Holtz-Neiderer K., Mohler M.J., Wendel C.S., Nixon B.P., Boulton A.J.M. (2004). Variability in Activity May Precede Diabetic Foot Ulceration. Diabetes Care.

[15] LeMaster J.W., Mueller M.J., Reiber G.E., Mehr D.R., Madsen R.W., Conn V.S. (2008). Effect of Weight-Bearing Activity on Foot Ulcer Incidence in People with Diabetic Peripheral Neuropathy: Feet First Randomized Controlled Trial. Phys. Ther..

[16] Balducci S., Iacobellis G., Parisi L., Di Biase N., Calandriello E., Leonetti F., Fallucca F. (2006). Exercise Training Can Modify the Natural History of Diabetic Peripheral Neuropathy. J. Diabetes Complicat..

[17] Allet L., Armand S., de Bie R.A., Golay A., Monnin D., Aminian K., Staal J.B., de Bruin E.D. (2010). The Gait and Balance of Patients with Diabetes Can Be Improved: A Randomised Controlled Trial. Diabetologia.

[18] Morrison S., Colberg S.R., Parson H.K., Vinik A.I. (2014). Exercise Improves Gait, Reaction Time and Postural Stability in Older Adults with Type 2 Diabetes and Neuropathy. J. Diabetes Complicat..

[19] Kluding P.M., Pasnoor M., Singh R., Jernigan S., Farmer K., Rucker J., Sharma N.K., Wright D.E. (2012). The Effect of Exercise on Neuropathic Symptoms, Nerve Function, and Cutaneous Innervation in People with Diabetic Peripheral Neuropathy. J. Diabetes Complicat..

[20] Feldman E.L., Stevens M.J., Thomas P.K., Brown M.B., Canal N., Greene D.A. (1994). A Practical Two-Step Quantitative Clinical and Electrophysiological Assessment for the Diagnosis and Staging of Diabetic Neuropathy. Diabetes Care.

[21] Fedele D., Comi G., Coscelli C., Cucinotta D., Feldman E.L., Ghirlanda G., Greene D.A., Negrin P., Santeusanio F. (1997). A Multicenter Study on the Prevalence of Diabetic Neuropathy in Italy. Italian Diabetic Neuropathy Committee. Diabetes Care.

[22] Mannocci A., Di Thiene D., Del Cimmuto A., Masala D., Boccia A., De Vito E., la Torre G. (2010). International Physical Activity Questionnaire: Validation and Assessment in an Italian Sample. Ital. J. Public Health.

[23] Vistisen D., Andersen G.S., Hansen C.S., Hulman A., Henriksen J.E., Bech-Nielsen H., Jørgensen M.E. (2016). Prediction of First Cardiovascular Disease Event in Type 1 Diabetes Mellitus. Circulation.

[24] Colberg S.R., Sigal R.J., Yardley J.E., Riddell M.C., Dunstan D.W., Dempsey P.C., Horton E.S., Castorino K., Tate D.F. (2016). Physical Activity/Exercise and Diabetes: A Position Statement of the American Diabetes Association. Diabetes Care.

[25] Herder C., Roden M., Ziegler D. (2019). Novel Insights into Sensorimotor and Cardiovascular Autonomic Neuropathy from Recent-Onset Diabetes and Population-Based Cohorts. Trends Endocrinol. Metab..

[26] Ahn S., Song R. (2012). Effects of Tai Chi Exercise on Glucose Control, Neuropathy Scores, Balance, and Quality of Life in Patients with Type 2 Diabetes and Neuropathy. J. Altern. Complement. Med..

[27] Streckmann F., Zopf E.M., Lehmann H.C., May K., Rizza J., Zimmer P., Gollhofer A., Bloch W., Baumann F.T. (2014). Exercise Intervention Studies in Patients with Peripheral Neuropathy: A Systematic Review. Sports Med..

[28] Riddell M.C., Gallen I.W., Smart C.E., Taplin C.E., Adolfsson P., Lumb A.N., Kowalski A., Rabasa-Lhoret R., McCrimmon R.J., Hume C. (2017). Exercise Management in Type 1 Diabetes: A Consensus Statement. Lancet Diabetes Endocrinol..

[29] Alves A.J., Viana J.L., Cavalcante S.L., Oliveira N.L., Duarte J.A., Mota J., Oliveira J., Ribeiro F. (2016). Physical Activity in Primary and Secondary Prevention of Cardiovascular Disease: Overview Updated. World J. Cardiol..

